# In Situ Synchrotron X‐ray Diffraction Studies of the Mechanochemical Synthesis of ZnS from its Elements

**DOI:** 10.1002/chem.202101260

**Published:** 2021-07-09

**Authors:** Hilke Petersen, Steffen Reichle, Sebastian Leiting, Pit Losch, Wolfgang Kersten, Tobias Rathmann, Jochi Tseng, Martin Etter, Wolfgang Schmidt, Claudia Weidenthaler

**Affiliations:** ^1^ Department of Heterogeneous Catalysis Max-Planck-Institut für Kohlenforschung Kaiser-Wilhelm-Platz 1 45470 Mülheim an der Ruhr Germany; ^2^ College of Mechatronics and Control Engineering Shenzhen University Shenzhen 518060 China; ^3^ P02.1 Petra III Deutsches Elektronen Synchrotron (DESY) Notkestr. 85 22607 Hamburg Germany

**Keywords:** in situ investigations, kinetic study, material synthesis, mechanochemistry, synchrotron study

## Abstract

Mechanochemistry, as a synthesis tool for inorganic materials, became an ever‐growing field in material chemistry. The direct energy transfer by collision of the educts with the milling media gives the possibility to design environmental‐friendly reactions. Nevertheless, the underlying process of energy transfer and hence the kinetics of mechanosynthesis remain unclear. Herein, we present in situ synchrotron X‐ray diffraction studies coupled with pressure measurements performed during the formation of ZnS and the subsequent phase transition (PT) from the hexagonal to the cubic modification. Milling Zn and S_8_ results in the sublimation of S_8_, observed by a sudden pressure increase. Simultaneously, the hexagonal metastable ZnS‐modification (wurtzite) forms. Via detection of the pressure maximum, the exact start of the wurtzite formation can be determined. Immediately after the formation of wurtzite, the structural PT to the thermodynamic stable cubic modification sphalerite takes place. This PT can be described by the Prout‐Tompkins equation for autocatalytic reactions, similar to thermally induced PT in sulfur vapor at high temperatures (T>1133 K). The increase in the reactivity of the wurtzite formation is explained by the reaction in sulfur vapor and the induction of defect structures by the collisions with the milling media.

## Graphical abstract

**A detailed in situ investigation of the mechanosynthesis** of the hexagonal ZnS modification (wurtzite) from its elements combining synchrotron powder diffraction and pressure measurements is presented. In addition, the subsequent phase transition from the hexagonal to the cubic structure (sphalerite) was investigated and the kinetics was explored.

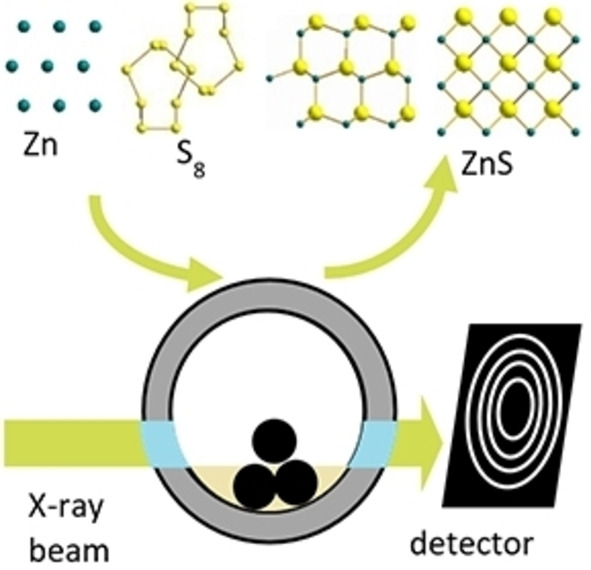

## Introduction

Mechanochemistry, which is already known since the stone age,^[1]^ is defined as the induction of a reaction via the direct absorption of the mechanical energy transferred from the milling media (reactor and balls) to the reactants.[^1^, ^2^] In particular, in recent years, mechanochemistry has become an ever‐growing field. One reason for the increasing interest is the potential to design highly cost‐and energy‐efficient as well as environmentally friendly chemical processes. This is realized by the described direct energy transfer without the use of any heat treatments or solvents.^[3]^ Removal of solvents is environmentally problematic and in addition, recovery rates usually do not exceed 50–80 %.^[2b]^ Because of these advantages, mechanochemical reactions are nowadays applied in various fields in chemistry to synthesize materials for applications, such as solid‐state hydrogen storage, catalysis, electrodes/ electrolyte for solid batteries, fuel cells, or functional ceramics.^[1b]^

The occurrence of the described local collision and frictional processes, their intensity, and relative importance for the milling process depends on the chosen milling conditions and the characteristic motion of the used mill type.^[4]^ The mill types mainly used in laboratories are planetary and shaker mills.^[4a]^ The milling principle of a planetary mill is based on two rotations; rotation of a disk and the opposite rotation of the milling jar (also called “reactor”) around its axis. This results in a primary movement of the ball on the reactor walls and a secondary movement across the reactor followed by a collision with the reactor walls.^[4a]^ Shaker mills work with displacement in the horizontal or vertical plane. The Retsch MM400 used here displaces the milling jar horizontally in an angular harmonic manner. Here, the ball alternately collides with the opposite reactor walls.^[4a]^ Consequently, in planetary mills shear forces are dominating while in shaker mills impact forces are effective.

Nevertheless, both types of mills transfer the mechanochemical energy locally, while in temperature‐induced reactions the energy is supplied to the bulk of the reactants.^[1b]^ Most models for the description of mechanochemical reactions were developed for inorganic systems.[^1b^, ^2b^, ^4a^] The two most common models are the hot spot and the magma‐plasma model.[^2b^, ^5^] The hot spot theory meanly considers frictional processes with plastic deformations resulting in local (∼1 μm^2^) temperatures of >1000 °C for a short period of time (10^−3^–10^−4^ s).^[2b]^ The magma‐plasma model, on the other hand, is based on the direct impacts. At the collision points, temperatures above 10^4^ °C can be generated, associated with transient plasmas and the ejection of energetic species including free electrons.[^2b^, ^6^] These models are not suitable to describe mechanisms involved in the synthesis of molecular organic compounds or metal‐organic framework materials. For such reactions, an extensive decomposition reaction would be expected.[^1b^, ^2b^] However, local heating, possible eutectic melting, the generation of new surfaces as well as of defects, improved contacts between solids and diffusing atoms can be considered effective for all mechanically synthesized solids.[^1b^, ^3a^, ^7^] This results in different reaction pathways to those observed in classical reactions (temperature‐ or pressure‐induced).^[3d]^ The tetragonal α‐PbO transforms upon thermal annealing to the orthorhombic β‐PbO around 763–813 K,^[8]^ while by heating β‐PbO to 673 K no evidence for a phase transition to α‐PbO is observed even after 24 h.^[9]^ During ball milling, no phase transformation of α‐ to β‐PbO is observed, whereas the phase transformation from β‐ to α‐PbO can be detected already at room temperature in both planetary and shaker mills.[^4b^, ^10^] The phase transition of β‐ to α‐PbO is even observed upon grinding in a mortar.^[9]^ CaCO_3_ crystallizes in three polymorphic structures, the metastable hexagonal vaterite, the low temperature‐stable orthorhombic aragonite, and the hexagonal calcite.^[11]^ Thermally, both vaterite (T=676–769 K) and aragonite (T=730 K) transform to calcite as the most stable phase. Mechanical treatment of vaterite results in a complete phase transformation to calcite as expected. However, upon further milling calcite reacts to aragonite quite in contrast to the thermal treatment.^[4b]^ Another example of significant differences between thermal and mechanical processes is ZnS, which crystallizes in two structure modifications,^[12]^ the thermodynamic stable cubic sphalerite[^12^, ^13^] and the high temperature‐stable hexagonal wurtzite phase.[^12^, ^13b^, ^14^] If classically synthesized via chemical vapor deposition, solid‐state reaction from a melt, or electrochemically, the formation of the cubic modification is observed.[^3b^, ^15^] Besides, solvent‐based synthesis, as precipitation from solution and hydrothermal treatment leads to the formation of the cubic modification.[^3b^, ^15^] The hexagonal wurtzite structure can be directly obtained only by hydrothermal synthesis in presence of thioglycolic acid as a stabilizing agent.^[16]^ In contrast to this, mechanical synthesis of ZnS from zinc acetate and sodium sulfide^[17]^ or directly from its elements^[3b]^ results in the formation of the hexagonal modification of ZnS though. By choosing either the thermal or the mechanical synthesis approach, different polymorphs are obtained, showing once again the incomparability between the two synthesis approaches. The cubic sphalerite can be reversibly transformed to the hexagonal modification by applying temperatures between 1230–1423 K,[^10a^, ^13b^, ^18^] or pressure of 1 GPa and 523 K.^[12]^ The kinetics of this phase transformation can be enhanced by the presence of zinc and sulfur vapors.^[19]^ Zinc vapors cause an increase in the reaction rate by enhancing the diffusion rate of zinc.^[19]^ However, the reaction still follows a first‐order kinetic. On contrary, performing the reaction in sulfur vapor can be either described by second‐order kinetics (T=1073–1113 K) or by the Prout‐Tompkins equation (T=1133–1173 K) depending on the applied temperature.^[19]^ The change in the kinetics of the reaction results from the enhanced adsorption of sulfur on the solid surface.^[19]^ Ex situ investigations of mechanochemical conversion with a planetary activator mill show a phase transition of wurtzite to sphalerite, but no evidence for a phase transition of sphalerite to wurtzite is observed.^[10a]^ Consistently, molecular dynamics simulations predict an irreversible phase transformation of wurtzite to sphalerite upon compression.^[12]^ The differences in thermal and mechanical behavior are likely caused by the different energy transfer mechanism from the milling media to the reactants.^[10a]^ While thermally induced energy activates all atoms uniformly throughout the bulk material,^[12]^ mechanical activation affects the solid locally and mainly at the surface, causing inelastic deformation at the collision point serving as nucleation center.^[12]^ Despite the growing interest and new in situ and operando studies,^[20]^ fundamental insights in the nature of mechanochemical processes and consequently the differences of thermal and mechanical activation are still missing.

The direct synthesis of ZnS from its elements is a simple inorganic model system. Depending on whether a mechanochemical or a conventional thermal synthesis route is chosen, different polymorphs are obtained. In addition, structural phase transformations have been shown to be depending on the synthesis route. Such, in situ X‐ray powder diffraction *(*XRPD) investigation on this model system might lead to new insights into the mechanical process. Here we report on the frequency dependency of the direct synthesis of ZnS from its elements. The coupling of in situ synchrotron XRPD with pressure measurements provides a fast way to monitor the crystallization of ZnS. The subsequent structural phase transformation upon further milling was studied via in situ synchrotron XRPD analyses. In situ XRPD analyses enable real‐time measurements^[20b]^ and thus the study of the underlying reaction kinetics of the transformation of wurtzite to sphalerite.

## Results and Discussion

**Figure 1:**
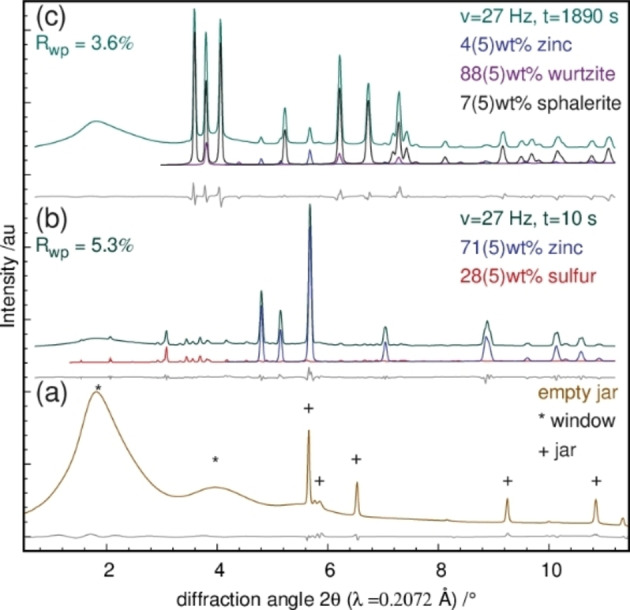
(a) XRPD data obtained from the milling vessel plus milling media milled at 27 Hz. The Rietveld plots of the reaction mixtures milled for (b) 10 s and (c) 1890 s are shown with the measured data (green), the difference curve (grey) and the refined phases (zinc (blue), sulfur (red), wurtzite (purple) and sphalerite (black)). The results of the quantitative phase analysis of the crystalline components are given in wt %.

For all in situ XRPD data, quantitative Rietveld refinements were performed. For structure refinements, the crystal structure data of zinc,^[21]^ sulfur,^[22]^ wurzite,^[23]^ and sphalerite^[24]^ were used. The Rietveld plots of a selection of refined in situ diffraction data sets and the diffraction pattern of an empty jar are shown in Figure 1. Detailed Rietveld plots of both in situ diffraction data sets can be found in the Supporting Information (Figure S1+S2). The pattern (a) shows the scattering contribution from the polymeric window material and the steel ball collected from an empty jar. The quantitative Rietveld refinement of the data obtained after 10 s (Figure 1b) of milling the educts at frequencies of 27 Hz reveals a mixture of 71(5) wt % metallic Zn and 28(5) wt % of solid S_8_. The additional set of reflections (phase fraction ∼1 wt %) belong to the steel vessel as well as the used steel ball. The significantly lower amount of solid S_8_ in the solid reaction mixture implies the evaporation of S_8_ by the mechanical impact.

**Figure 2:**
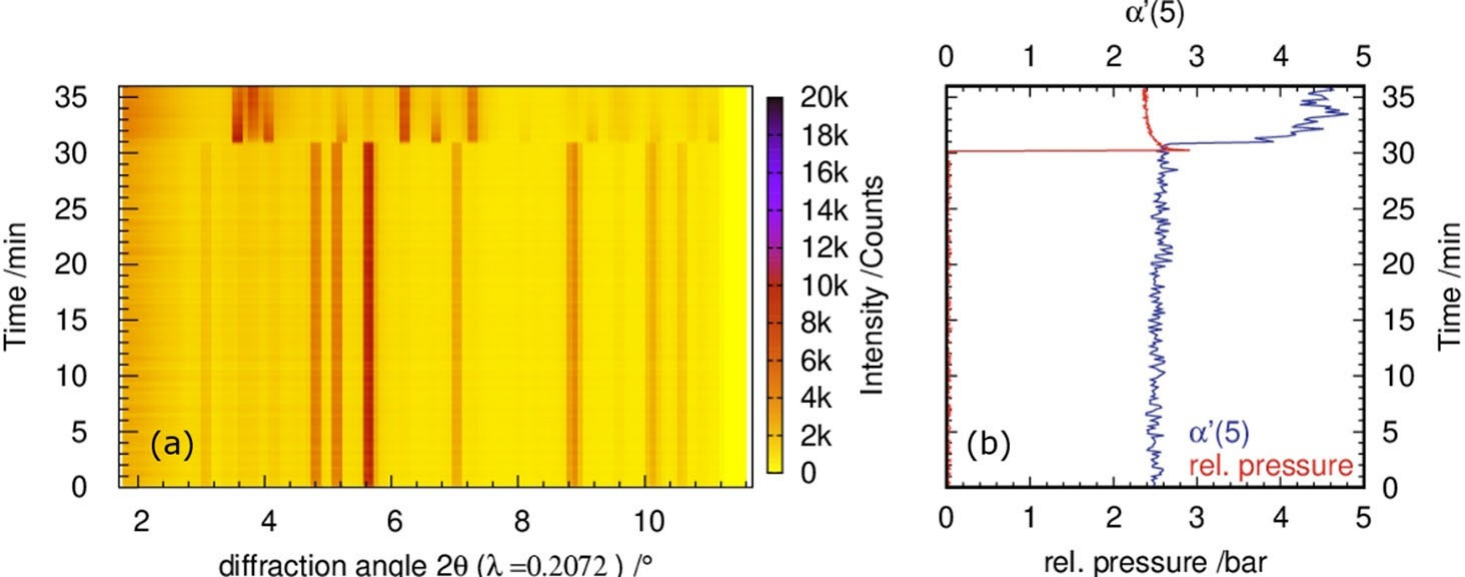
a) Time‐dependent in situ XPRD synchrotron data along with (b) the corresponding evolution of the autocorrelation parameter α’(5) and pressure while milling the 1 : 1 Zn/S_8_ mixture at 27 Hz.

To be able to observe possible pressure changes inside the jar during ball milling, a pressure sensor was connected to the milling jar. Monitoring the relative pressure of a reaction mixture milled with a frequency of 27 Hz for 40 min shows a sudden pressure increase with a maximum after 30 min 20±10 s (Figure 2b). This is followed by a decay of the pressure during further milling. At the relative pressure maximum, the simultaneous disappearance of S_8_ reflections and an increase in the autocorrelation function parameter α’(5) (t_max_=31 min) as well as the appearance of new reflections with the main reflection around 3.5–4.1° 2θ can be observed (Figure 2a+b). The autocorrelation function is explained in the experimental section.^[25]^ The new reflections show the formation of hexagonal ZnS (wurtzite) crystallizing in space group $P6_{3}\mathrm{mc}$ .^[24]^ In situ pressure measurements of Cu/S_8_‐mixtures in molar ratios from 1 : 1 to 3 : 1 by Baláž et al. showed similar pressure increases upon milling.^[26]^ Via ex situ XRPD measurements, the authors could relate these pressure increases to the formation of CuS. Here, the combination of in situ XRPD measurements and monitoring the pressure in the milling vessel (Figure 2b) shows that the ZnS formation from elemental sulfur and zinc in the ball mill is a heterogeneous process, where gaseous sulfur reacts with solid zinc. After the formation of ZnS, the relative pressure inside the reaction‐vessel does not decrease to zero but tends to a value of 2.3±0.2 bar. The observed residual relative pressure indicates that unreacted sulfur remains in the gas phase. In agreement with that, not all of the metallic zinc in the 1 : 1 Zn/S‐mixture is converted to ZnS and the remaining zinc can be detected (Figure 2a). The residual educts indicate that for the ZnS‐formation a partial sulfur pressure above 2.3 bar is required. However, the combination of in situ XRPD and pressure measurements shows that it is possible to correlate the pressure maximum inside the jar with the start of the formation of ZnS. Based on this relation, a fast detection of the reaction start even without synchrotron radiation data is possible.

**Figure 3:**
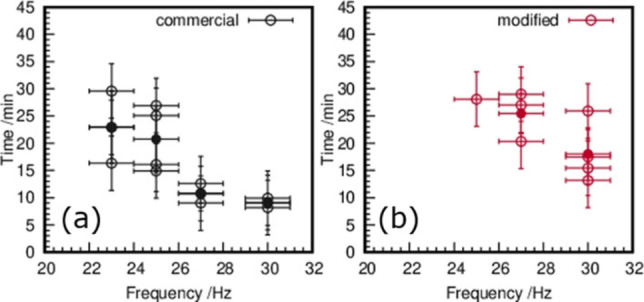
(a) Times at which relative pressure maxima at different frequencies are observed with commercial jar holders (open black circles) together with their average value (filled black circles) against the applied frequencies. (b) Times at which relative pressure maxima at different frequencies are observed with the modified jar holders (open red circles) along with the respective average (filled red circles) against the applied frequencies.

The detection of the relative pressure maximum allows the investigation of the dependence of the crystallization start and the applied milling‐frequencies (ν=23, 25, 27, 30 Hz) (Figure 3). Each experiment was repeated five times. Not in all experiments, a relative pressure maximum indicating a reaction to ZnS could be observed. This shows the reduced reproducibility of the conducted reactions. Figure 3 shows the times when the pressure maximum was observed for different frequencies. For a first series of experiments, the commercial jar holders (Figure 3a) were used. For in situ synchrotron experiments, the jar holders need to be extended in such a way that the jars are lifted into the X‐ray beam. A second series of milling experiments were performed with the modified holders (Figure 3b), which places the milling jar above the mill.^[27]^ Both experimental series imply a decrease in the induction period before crystallization starts with the increase of the milling frequency. Using the commercial jar holder, a reaction in the frequency range from 23–30 Hz can be observed. For lower frequencies (*v*≤25 Hz), the start of the crystallization is retarded, compared to the crystallization at higher frequencies. For the modified jar holder, no reaction was observed at 23 Hz. Compared to the start of crystallization with the commercial jar holders, the crystallization is delayed at all used frequencies *v*≤25 Hz for the modified jar holder. These results imply a lowering in the energy supply in the case of using the modified jar holder.

**Figure 4:**
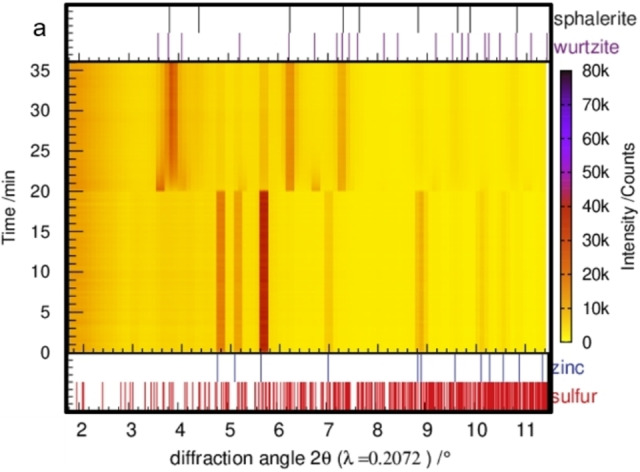
In situ XRPD pattern of a 1 : 1 reaction mixture of zinc and sulfur milled with 30 Hz.

In situ XRPD data were collected for varying frequencies (ν=23 Hz, 25 Hz, 27 Hz, 30 Hz). In all experiments, the formation of hexagonal ZnS was observed. In Figure 4, the collected in situ data of a stoichiometric Zn/S mixture milled at 30 Hz is exemplarily shown. The diffraction data show neither an increase of the amorphous background nor any shift of the reflections, indicating no amorphization of the sample nor formation of intermediate compounds during the synthesis of ZnS from the elements. The fast formation of ZnS is consistent with the results obtained from the combined in situ pressure and XRPD measurement, implying a fast heterogeneous process of gaseous sulfur and solid zinc forming ZnS. These results are contradictory to the observations made by Baláz et al.^[28]^ The authors observed an initial amorphization step when milling a 1 : 2 Zn/S mixture in a planetary mill (AGO‐2; V.R.F. Technology, Hungary). Different major forces in the different mill types may explain the observed differences in the ZnS formation. In the planetary mills, the energy transfer proceeds mainly via shear forces, while in the shaker mill direct impact of the balls is dominating.^[4]^ In addition, differences in the milling conditions, such as ball diameter, ball‐to‐powder ratio, and vessel volume, alter the milling mechanism.

**Figure 5:**
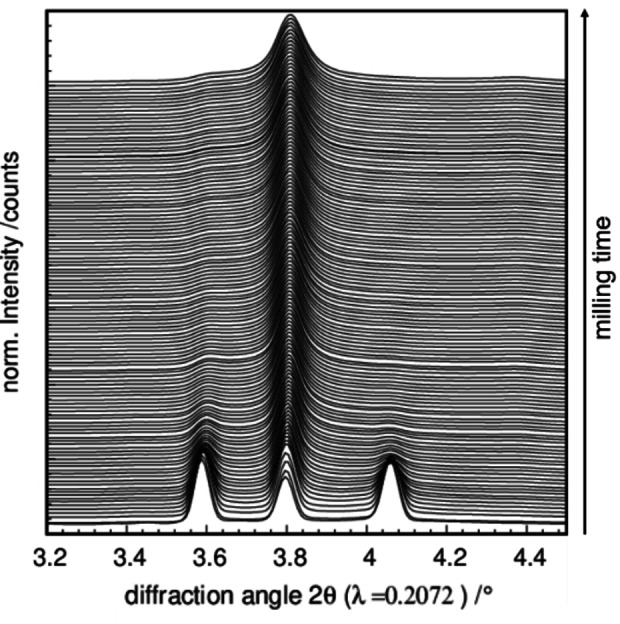
Evolution of the main reflections of the hexagonal ZnS‐modifications upon milling with 30 Hz.

The changing intensities of the three main reflections of the wurtzite structure in the measuring range from 3.5–4.1° 2θ indicate an immediate phase transformation of the metastable hexagonal ZnS modification to the cubic one (Figure 5). Both ZnS modifications, wurtzite and sphalerite, have a reflection at the middle position (∼3.8° 2θ), while the two reflections at 3.6° 2θ and 4.1° 2θ are typical for the wurtzite structure. Hence, the phase transformation from wurtzite to sphalerite can be followed by the intensity ratios of these three reflections. The nature and in particular kinetics of the phase transformation is further investigated via in situ diffraction experiments with varying milling‐frequencies.

**Figure 6:**
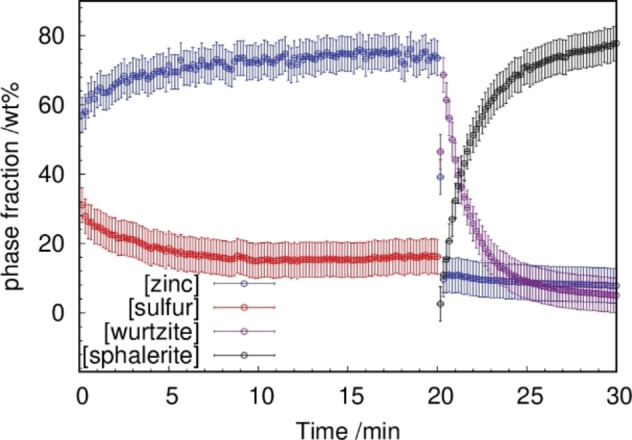
Phase fractions (wt %) of the crystalline phases determined by Rietveld refinements of data obtained during ball milling at 30 Hz.

Quantitative phase analyses were performed for all in situ data. Figure 6 shows exemplarily the phase fraction analysis of a sample milled at 30 Hz. Starting from a stoichiometric mixture of Zn and S, the quantitative Rietveld refinement revealed a significantly lower sulfur content as expected (also compare Figure 1). The formation of wurtzite is detected only after ∼20 min milling time. The instant increase of the wurtzite content visualizes the fast, one‐step formation reaction to the wurtzite structure. However, the phase transformation from wurtzite (46(1) wt %) to sphalerite (3(1) wt %) proceeds immediately. The phase fraction of sphalerite increases very quickly and levels out after ∼22 min. Afterwards, the phase fraction stays constant at a value ∼80 wt %. It is known that the mechanical activation of solids by mechanical processes affects the reaction kinetics. The reaction temperatures are lowered and the reaction rates increase.[^3a^, ^12^, ^13b^] The mechanical impact on solids results in an increase of the defect density and, consequently, in higher mobility of atoms.[^1b^, ^3a^, ^13b^] In addition, the observed sublimation of sulfur from the reaction mixture causes a homogeneous distribution of sulfur and thus results in an increased reactivity. For the phase transformation of wurtzite to sphalerite in sulfur vapors at different temperatures, an enhancement of the reactivity was reported by Bansagi et al.^[19]^ The authors distinguish two temperature regimes. Between 1133 K and 1153 K, the reaction is described by second‐order kinetics and at higher temperatures (T≥1133 K) with the Prout–Tompkins model for autocatalytic reactions. According to Bansagi et al., the change of the kinetic model as well as the enhancement of the reaction is caused by the increase of seed crystals on the surface of wurtzite crystals.^[19]^

**Figure 7:**
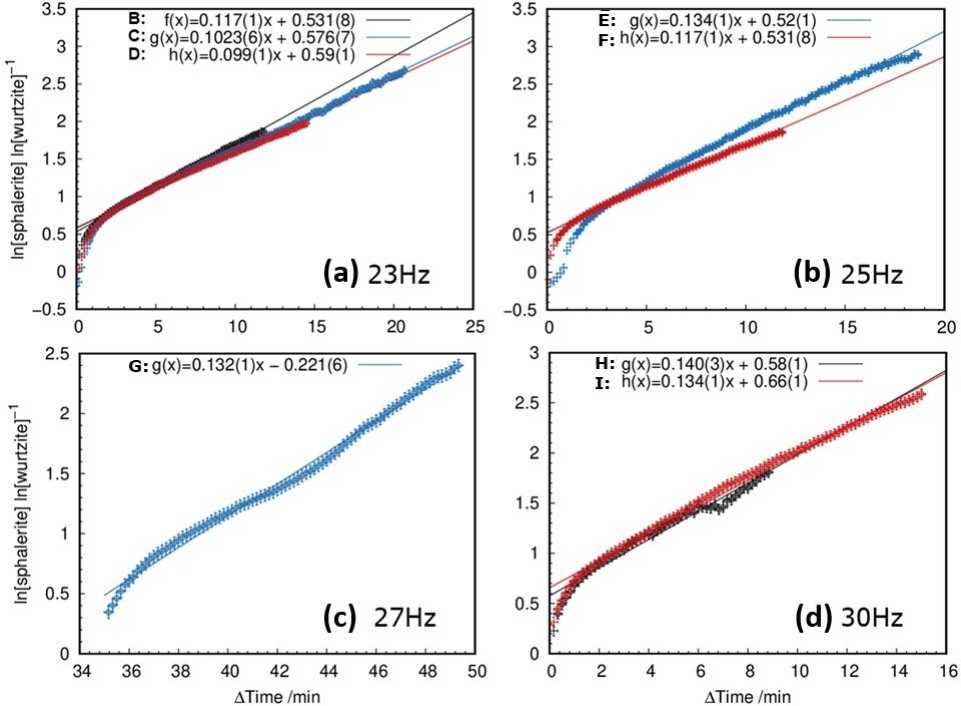
Linear regression curves of the obtained ratio of the logarithmic phase fractions of the two ZnS modifications for different frequencies 23 Hz (a), 25 Hz (b), 27 Hz (c) and 30 Hz (d).

The Prout‐Tompkins model for autocatalytic reactions can describe the mechanical induced phase transition of wurtzite to sphalerite in the frequency range of 20–30 Hz. The Prout–Tompkins model was derived for the reaction at constant temperatures and with a constant energy supply. The milling with a constant frequency can be assumed to result in a globally constant energy supply. In solid state reactions, autocatalysis occurs if the nuclei growth promotes the reaction due to the induction of imperfections at the reaction interface.^[29]^ The termination occurs when the reaction propagates into the educt.^[29]^ In the presented study, a comparable reaction mechanism to the thermal investigation by Bansagi et al. is assumed.^[19]^ Both the increased defect concentration induced by the collisions with the milling media and gaseous sulfur atmosphere enhance the atomic diffusion on the surface. This results in an enhancement of nuclei formation on the crystallite surface which propagates into the crystal via the formation of defects in the crystal structure. The logarithmic ratio of sphalerite to wurtzite versus the milling time shows a linear dependence after the reaction start (Figure 7). From the linear part of the curves, reaction rates were obtained via linear regression (Table 1). As expected, the reaction rates increase with the applied milling‐frequencies, reflecting the higher acceleration of the milling balls and resulting in higher energy transfer to the reactants. In addition, the number of collisions of the milling media with the reactants is enhanced, presumably resulting in a higher defect concentration in the solid reactants. Higher defect concentration then likely causes improved atomic diffusion.^[1b]^

**Table 1:** Reaction rates (*k*) obtained from linear regression of the logarithmic phase fraction of the two ZnS modifications.

Sample name	Experimental conditions (frequency; time per frame)	*k*	*k*_average_
B	23 Hz; 10 s	0.117(1)	0.106(7)
C	23 Hz; 10 s	0.1023(6)	
D	23 Hz; 10 s	0.099(1)	
E	25 Hz; 10 s	0.134(1)	0.125(8)
F	25 Hz; 10 s	0.117(1)	
G	27 Hz; 10 s	0.131(1)	0.131(1)
H	30 Hz; 10 s	0.140(1)	0.142(7)
I	30 Hz; 10 s	0.134(1)

**Figure 8:**
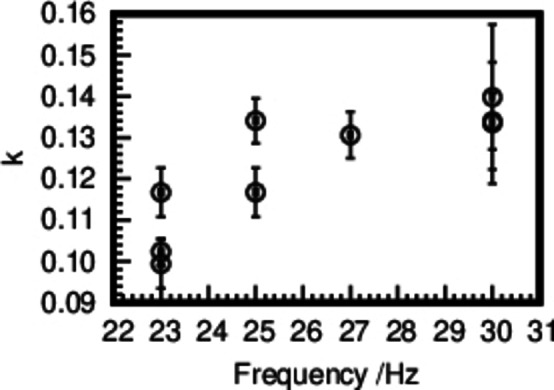
Obtained reaction rates plotted versus applied frequencies

Plotting the obtained reaction rates *k* versus the frequency (Figure 8) shows a positive relation. Assuming that the complete kinetic energy of the milling media is transferred to the reactant during milling, the relation hints at a strong dependence of the reaction rate on the velocity of the milling media.^[30]^ The obtained reaction rates show a high variety, especially at lower frequencies. The mechanochemical process is influenced by several factors, such as the ball‐to‐powder ratio, the material the milling media is made of, the volume of the reaction jar, and the milling frequency.^[13b]^ The velocity and as such the impact power of the grinding media depends on the acceleration of the reaction jar as well as the exact mill geometry. The presented in situ experiments were conducted over a time range of several months. During this time the mill, in particular its bearings might have aged, reducing the reproducibility of the experiments. A shaker mill displaces the jars in an angular harmonic movement in the horizontal plane, describing a radial movement. The radius of the movement affects the impact power of the milling media. For the discussed experiments, the geometry of the mill was changed in order to enable the positioning of the jars in the X‐ray beam for in situ synchrotron measurements. Already slight geometric changes affect both the impact of the milling balls, as well as the reproducibility of the experiments.

## Conclusion

The metastable wurtzite structure can be synthesized via mechanochemistry in a shaker mill in an enormously fast reaction. Unlike in a planetary mill, no amorphous intermediate was observed,^[3a]^ showing once again that the milling processes in different types of mills are not directly comparable. While in the planetary mill shear forces are dominating, direct impact forces of the milling media with the reactants are causing the reactions in a shaker mill. The fast formation rate of ZnS can be explained by the sublimation of sulfur through the direct impact of the milling media. This was proven via the coupling of pressure measurements and in situ synchrotron diffraction experiments. Furthermore, the assumed induction of defects as well as the formation of new surfaces in the solid zinc might enhance the formation of seed crystals on the zinc surface. Via the sudden pressure changes, the ZnS formation can be detected very fast. The ZnS formation shows a positive dependence on the applied milling frequency. In addition, a reduction of the energy supply by the modified jar holders for placing the jars in the X‐ray beam could be observed. Noteworthy is the enhanced reproducibility of experiments at higher frequencies, assumingly due to the quick equilibration of the milling conditions.

Immediately after the formation of hexagonal ZnS, a phase transformation to the thermodynamic stable cubic ZnS is observed. The phase transformation can be described by the Prout–Tompkins kinetics for autocatalytic reactions. Assumingly, the induced defect concentration increases the atomic diffusion and thus accelerates the phase transformation. Hence, an enhanced reactivity via milling can be assumed, caused by the sublimation of the sulfur and by the formation of new surfaces and increased defect density in the solid reactants.

The relation between the applied frequency and the obtained reaction rate implies a string effect of the velocity of the milling media on the reaction. However, the obtained reaction rates show low precisions, resulting from several factors. These are minute variations of milling conditions, such as ball‐to‐powder ratio, aging (wearing) of the reactor, jar holder and mill. In addition, slight changes in the geometry of the mill as modification of the holders to place the jar in the X‐ray beam influences the energy supply. The experimental setup determines the exact frequency and so the impact power and as such the energy transfer to the reactant. All these parameters must be thoroughly taken into account for the evaluation of experimental data from milling experiments as illustrated in this work.

## Experimental Section

**Figure 9:**
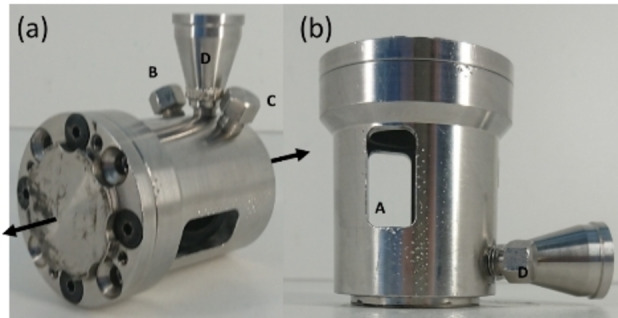
Design of the milling jar for in situ XRPD measurements (horizontal view (a), side view (b)) equipped with an X‐ray transparent window (A), gas inlet (B+C) and a gas outlet (D). The black arrows mark the horizontal motion of the vessel.

All mechanical experiments were carried out with a RETSCH MM400 shaker mill (Retsch GmbH Haan, Germany). The mill was equipped with modified reaction jar holders, which enable the positioning of the milling jars into the X‐ray beam on a diffraction instrument at the synchrotron beamline.^[27]^ The stainless steel milling jars with an inner volume of 25 mL were specially designed for in situ measurements (Figure 9a+b). In contrast to commercially available milling jars, the jars constructed at the Max‐Planck‐Institut für Kohlenforschung are equipped with two X‐ray transparent polymer windows. The cavities in the steel body are covered with a polymeric ring‐inlet forming a smooth inner surface of the reaction container. These windows allow the X‐ray beam to pass through the jars, but they also contribute to the overall diffraction pattern. For future gas experiments, in‐ and outlet lines enable the use of different gas atmospheres during milling. For the mechanochemical synthesis, metallic zinc powder (Merck, CAS: 7440‐66‐6) and sulfur powder (Sigma‐Aldrich, CAS: 7704‐34‐9) were mixed in a molar ratio of 1 : 1. 1 g of the reaction mixture was filled into the stainless steel milling jars. One 15 mm steel ball (m_ball_=13.6 mg) was used for milling. Different milling frequencies (ν=23, 25, 27, 30 Hz) were applied (Table 2). The milling time varied between 30 to 45 min depending on the progress of the reaction. For the measurement of the pressure evolution inside the milling jar during milling, a pressure sensor (Jumo GmbH & Co.KG Fulda, Germany) was connected via a polyamide tube with the milling vessel.^[31]^ Thereby the polyamide tube was connected via a funnel, the increasing diameter causes a tortuous flow which prevents the reactants to enter the polyamide tube. In addition to the synchrotron experiments, conventional in‐house (ex situ) milling experiments were repeated 5 times for each frequency with and without the modified reaction holders designed to place the reaction container above the mill.

**Table 2:** Overview of the experimental conditions of all in situ XRPD measurements.

Sample name	Milling conditions (frequencies and ball)		Measuring conditions
A	20 Hz:	1*ball 15 mm	10 s/frame
B	23 Hz:	1*ball 15 mm	10 s/frame
C	23 Hz:	1*ball 15 mm	10 s/frame
D	23 Hz:	1*ball 15 mm	10 s/frame
E	25 Hz:	1*ball 15 mm	10 s/frame
F	25 Hz:	1*ball 15 mm	10 s/frame
G	27 Hz:	1*ball 15 mm	10 s/frame
H	30 Hz:	1*ball 15 mm	10 s/frame
I	30 Hz:	1*ball 15 mm	10 s/frame

The in situ investigations were performed at P02.1 (Petra III DESY, Hamburg, Germany) using the experimental setup as described above.^[32]^ The experimental parameters are summarized in Table 2. The data were collected with a Perkin Elmer XRD1621 CN3‐EHS detector (200×200 μm^2^ pixel size, 2048×2048 pixel area) or a Varex XRD 4343CT (150×150 μm^2^ pixel size, 2880×2880 pixel area) detector. All data were collected using a wavelength of λ=0.2072 Å. X‐ray powder diffraction (XRPD) patterns were recorded with a counting rate of 10 s per frame. The obtained data were integrated with Dawn 2.6.0 (Diamond Light Source Ltd, Oxfordshire, United Kingdom).^[33]^ All measurements were evaluated with the Rietveld software Topas 6 (Bruker AXS GmbH, Karlsruhe, Germany).^[34]^ The implemented fundamental parameter approach was used for the description of the diffractometer profiles, determined using a Si standard reference material. Additionally, the autocorrelation parameter [Eq. 1]:

(1) $$\alpha^{{}^{'}}\left(\omega^{{}^{'}}\right)=\;\sqrt{\frac{\omega^{{}^{'}2}}{-4\ln({\mathrm{Corr}}_{\mathrm{Norm}}\left(g\left(\omega \right),g\left(\omega \right),\omega^{{}^{'}}\right))}}$$

was calculated from individual data sets as a global parameter (ω′=5) was used.^[25]^ With ω’ is a shift parameter applied to the primary data and g(ω) is a Gaussian function to describe the peak shape of the autocorrelation function. Via the autocorrelation function, a single parameter for each data set can be derived which is highly sensitive to changes in the number of reflections as well as their widths. Such the autocorrelation parameter may be used to identify the start of reactions.^[35]^

## Conflict of interest

The authors declare no conflict of interest.

## Supporting information

As a service to our authors and readers, this journal provides supporting information supplied by the authors. Such materials are peer reviewed and may be re‐organized for online delivery, but are not copy‐edited or typeset. Technical support issues arising from supporting information (other than missing files) should be addressed to the authors.

Supporting Information
