# Supplementary material for: In Situ Synchrotron X‐ray Diffraction Studies of the Mechanochemical Synthesis of ZnS from its Elements
Source: Chemistry. 2021 Jul 9;27(49):12558–65. doi: 10.1002/chem.202101260 (PMC8456871; doi:10.1002/chem.202101260)
Supplement: Supplementary file 1 — Supporting Information [file CHEM-27-12558-s001.pdf]

Chemistry–A European Journal

Supporting Information

In Situ Synchrotron X-ray Diffraction Studies of the Mechanochemical Synthesis of ZnS from its Elements

Hilke Petersen, Steffen Reichle, Sebastian Leiting, Pit Losch, Wolfgang Kersten, Tobias Rathmann, Jochi Tseng, Martin Etter, Wolfgang Schmidt, and Claudia Weidenthaler*

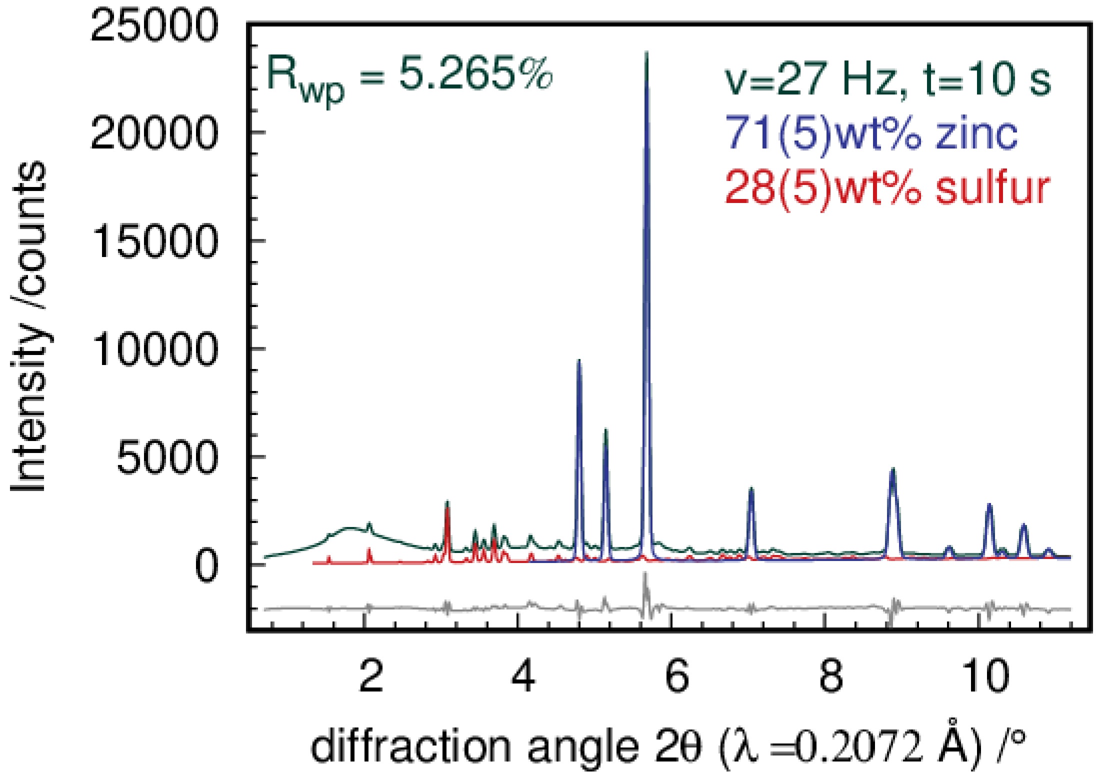

Figure S1: The Rietveld plot of the reaction mixtures milled with 27 HZ for 10 s are shown with the measured data (green), the difference curve (grey) and the refined phases (zinc (blue), sulfur (red), wurtzite (purple) and sphalerite (black)). The results of the quantitative phase analysis of the crystalline components are given in wt%.

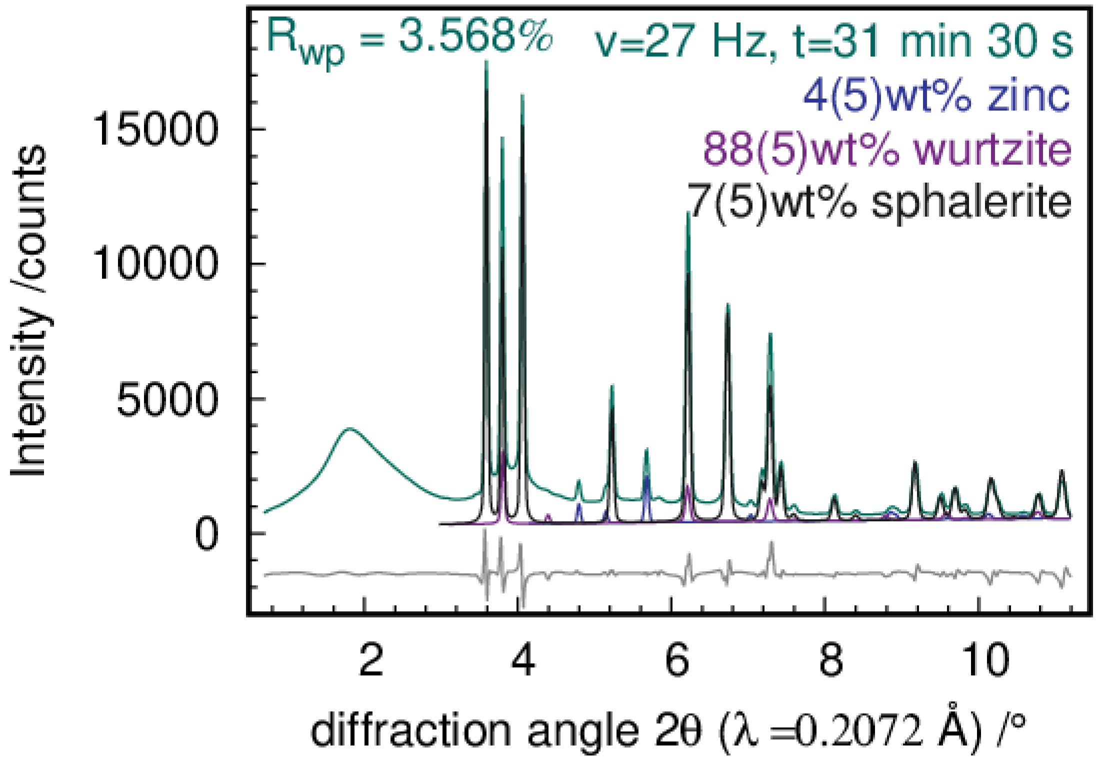

Figure S2: The Rietveld plot of the reaction mixtures milled with 27 HZ for 31 min 10 s are shown with the measured data (green), the difference curve (grey) and the refined phases (zinc (blue), sulfur (red), wurtzite (purple) and sphalerite (black)). The results of the quantitative phase analysis of the crystalline components are given in wt%.
